# Drug Delivery Strategies for Enhancing the Therapeutic Efficacy of Toxin-Derived Anti-Diabetic Peptides

**DOI:** 10.3390/toxins12050313

**Published:** 2020-05-10

**Authors:** Reeju Amatya, Taehoon Park, Seungmi Hwang, JaeWook Yang, Yoonjin Lee, Heesun Cheong, Cheol Moon, Hyun Duck Kwak, Kyoung Ah Min, Meong Cheol Shin

**Affiliations:** 1 College of Pharmacy and Research Institute of Pharmaceutical Sciences, Gyeongsang National University, 501 Jinju Daero, Jinju, Gyeongnam 52828, Korea; reejuamatya94@gmail.com (R.A.); xornf@naver.com (T.P.); 2 College of Pharmacy and Inje Institute of Pharmaceutical Sciences and Research, Inje University, 197 Injero, Gimhae, Gyeongnam 50834, Korea; hsm8549@naver.com; 3 Department of Ophthalmology, Busan Paik Hospital, Inje University College of Medicine, 75 Bokjiro, Busanjin-gu, Busan 47392, Korea; oculoplasty@gmail.com (J.Y.); jordan90@hanmail.net (H.D.K.); 4 T2B Infrastructure Center for Ocular Disease, Inje University Busan Paik Hospital, 81 Jinsaro 83 Beon-gil, Busanjin-gu, Busan 47397, Korea; 5569sy@naver.com; 5 Division of Cancer Biology, National Cancer Center, 323 Ilsan-ro, Ilsandong-gu, Goyang, Gyeonggi-do 10408, Korea; heesunch@ncc.re.kr; 6 College of Pharmacy, Sunchon National University, 255 Jungang-ro, Suncheon, Jeonnam 57922, Korea; cheolm@scnu.ac.kr

**Keywords:** toxin, diabetes, anti-diabetic peptide, drug delivery, plasma half-life, cell-penetrating peptide

## Abstract

Toxin peptides derived from the skin secretions of amphibians possess unique hypoglycemic activities. Many of these peptides share cationic and amphipathic structural similarities and appear to possess cell-penetrating abilities. The mechanism of their insulinotropic action is yet not elucidated, but they have shown great potential in regulating the blood glucose levels in animal models. Therefore, they have emerged as potential drug candidates as therapeutics for type 2 diabetes. Despite their anti-diabetic activity, there remain pharmaceutical challenges to be addressed for their clinical applications. Here, we present an overview of recent studies related to the toxin-derived anti-diabetic peptides derived from the skin secretions of amphibians. In the latter part, we introduce the bottleneck challenges for their delivery in vivo and general drug delivery strategies that may be applicable to extend their blood circulation time. We focus our research on the strategies that have been successfully applied to improve the plasma half-life of exendin-4, a clinically available toxin-derived anti-diabetic peptide drug.

## 1. Introduction

Diabetes is a metabolic disorder in which the blood glucose level is not orderly regulated and maintains a higher-than-normal level. Specifically, type 2 diabetes mellitus (T2DM), the most prevalent type of diabetes, is heavily related to obesity, a prevalent condition in the modern lifestyle [1]. T2DM is characterized by high blood glucose levels, insulin resistance, and progressively reducing plasma insulin levels [2]. The conventional therapeutics for the T2DM usually work by inhibiting the glucose production in the liver, sensitizing the insulin receptors, and enhancing insulin secretion [3]. At the later stage of T2DM, when eventually the pancreatic beta cells become deteriorated and unable to produce sufficient insulin, treatment of exogenous insulin is necessary [4]. Considering the life-threatening complications of diabetes and the inability of current therapies to completely resolve the diabetic conditions, there has continuously been a need to discover a novel therapeutic agent [5].

To date, natural and synthetic peptides which are biological active are gaining great attention as potential drug candidates [6,7,8,9]. Compared with small molecule-based drugs, these therapeutic peptides are usually considered more potent and specific, but less toxic [10]. Moreover, thanks to advances in techniques for peptide synthesis, screening, and analysis, the industrialization of peptide drugs has become more feasible. In this context, exendin-4 (tradename: Byetta; Amylin Pharmaceuticals, Inc.), was approved by the FDA in 2005 as the first toxin-derived peptide therapeutic for the treatment of T2DM [11]. Exendin-4, a 39-mer peptide, was first identified in the salivary secretions of a venomous lizard species known as the Gila monster, *Heloderma suspectum* [11]. The exendin-4 has a 53% structural similarity to the GLP-1 (Glucagon-like protein-1) and possesses anti-diabetic activity [11]. One of the major advantages of exendin-4 over the endogenous GLP-1 is its great stability against the dipeptidyl peptidase-4 (DPP-4) enzyme that enables much longer action in the body [12,13]. However, the plasma half-life of exendin-4 is still relatively short (2.4 h) and thus requires twice-daily injections for adequate hypoglycemic control. To reduce the frequency of the injection and improve the patients’ compliance, an extensive amount of research has been conducted to develop effective ways to prolong the plasma half-life of exendin-4 and, indeed, some of those exendin-4 analogs are clinically available [14,15,16,17].

Following the success of exendin-4, many peptides with potent anti-diabetic activities have been discovered from various animal sources including cone snails [18], reptiles [19], and amphibians [20]. These peptides could stimulate insulin release from beta cells and lower blood glucose levels in animals. Their mechanism for the insulinotropic activities varies depending on the peptide. While the exendin-4 and the insulin toxin of cone snails elicit insulin release by binding to their specific receptors (GLP-1R and insulin receptor, respectively), various snake venoms directly act on channels that play major roles in insulin secretion [18,19,20]. However, for the toxin-derived anti-diabetic peptides derived from the skin secretions of amphibians, their molecular targets are not yet clear.

To date, a large group of toxin-derived anti-diabetic peptides have been discovered from the skin secretions of amphibians and their activities have been assessed. Recently, more and more studies have been focused on the modification of the peptide sequences and structures to reduce their intrinsic toxicities and improve glucoregulatory efficacy. However, due to drug delivery challenges, many studies are limited to cellular levels and a systemic characterization of their activities in animal models is as yet insufficient. This review will introduce toxin-derived anti-diabetic peptides focused on the peptides derived from the skin secretions of amphibians that possess cell-penetrating ability. After that, their pharmaceutical challenges will be discussed, followed by an overview of drug delivery strategies that may be applicable to extend their blood circulation time.

## 2. Toxin-Derived Anti-Diabetic Peptides from Skin Secretions of Amphibians

Skin secretions of amphibians contain various types of host defense peptides that possess a wide spectrum of activities in inflammation, infection, cancer, immunomodulation, and glucoregulation [21,22]. With the help of advances in peptidomimetic analysis techniques, to date, a number of peptides have been identified and a group of these host defense peptides have gained continuous attention due to their potential to be used as anti-diabetic drugs [20]. These toxin-derived anti-diabetic peptides generally share cationic and amphipathic properties in their structure and commonly exhibited insulinotropic actions in vitro and, in diabetic animal models, elicited hypoglycemic effects [22]. In some cases, hypolipidemic and anorexic effects were also frequently observed from the test animals [11,21]. The following are several representative examples of the toxin-derived anti-diabetic peptides from the skin secretions of amphibians. Their peptide sequences and activity levels are summarized in Table 1.

### 2.1. Esculentin-2CHa

Esculentin-2CHa is a 37-mer bioactive peptide isolated from the skin secretion of the Chiricahua leopard frog *Lithobates chiricahuensis* (Ranidae) [23,32]. The esculentin-2CHa possesses various activities including anti-tumor, anti-microbial, and anti-diabetic activity [23,24]. However, it could exert moderate cytotoxicity against erythrocytes (LC_50_: 150 μM). Increasing the cationicity of the peptides by amino acid substitution with cationic residues (Lys or Arg) may promote their interaction with cell membranes and subsequent internalization. In the case of esculentin-2CHa, Lys (K) substitutes showed enhanced bioactivities. For example, [L28K]esculentin-2CHa, an analog of esculentin-2CHa with a higher cationicity, could elicit enhanced glucose tolerance and insulin secretion in HFD (High fat diet)-fed mice [33]. The authors postulated that this may be caused by higher cell uptake of the [L28K]esculentin-2CHa. In another case study, Lys substitution of Asp20 or Asp27 of the esculentin-2CHa showed enhanced antimicrobial effects [24]. However, this augmented anti-microbial activity appeared to be correlated with increased toxic hemolytic activity. On the other hand, the deletion of the N-terminal domain (GFSSIF) or the cyclic C-terminal domain (CKISKQC) and substitution of Cys31 and Cys37 residues to Ser decreased the antimicrobial activity [24]. Specifically, the deletion of the cyclic C-terminal domain (CKISKQC) from the esculentin-2CHa allowed depletion of antimicrobial activity but had little effect on insulin-releasing activity [34]. This truncated esculentin-2CHa (esculentin-2CHa(1–30)) was shown to efficiently internalize BRIN-BD11 cells without disrupting the cell membranes and stimulate the release of insulin without indication of cytotoxicity. Interestingly, this may reflect the cell-penetrating peptide (CPP)-like property of the esculentin-2CHa(1-30). Different from the exendin-4 whose anti-diabetic activity occurs by binding to GLP-1R, most of the toxin-derived anti-diabetic peptides do not have known receptors for their action. Rather, it was thought that these peptides will enter the β cells (via a transduction mechanism like cell-penetrating peptides) and induce the exocytosis of insulin by a K_ATP_ channel-independent pathway. The proposed mechanism for the insulinotropic action by esculentin-2CHa(1-30) involves: (1) cell internalization of esculentin-2CHa(1–30), (2) K_ATP_-independent cell membrane depolarization, (3) increase of intracellular Ca^2+^ level, and (4) insulin exocytosis [35].

### 2.2. Tigerinin-1R

Tigerinins are cationic host defense peptides first identified in the Indian frog *Hoplobatrachus tigerinus* in the family of Dicroglossidae [25,36]. Among the tigerinins, the tigerinin-1R which was first isolated from the skin secretion of the African crowned bullfrog *Hoplobatrachus occipitalis* could elicit anti-diabetic activity [25]. Tigerinin-1R could stimulate insulin release from the BRIN-BD11 cells at concentrations above 0.1 nM [25]. The administration of tigerinin-1R to HFD-fed mice (twice administered with 75 nmol/kg by i.p. (intraperitoneal) injection for 15 days) produced a significant decrease of plasma glucose, glucagon and triglyceride levels, and an increase in the plasma insulin level [37]. The mechanism for the insulinotropic action of the tigerinin-1R also involved cell membrane depolarization and an increase in intracellular calcium concentrations. Notably, the tigerinin-1R showed no significant hemolytic activity (at concentrations up to 500 μM) in a short-term (60 min) in vitro study with human red blood cells [25]. Srinivasan et al. carried out a structure-activity study by relating the insulinotropic activity with the modification of the amino acids of tigerinin-1R [38]. In the study, Ser4, Ala5, Ile6, Leu8, Ile10 and His12 were substituted to Lys, Arg or Trp residues. Interestingly, while increasing the cationicity had little effect on the insulinotropic activity, increasing the hydrophobicity by substitution of the amino acids to Trp showed higher potency than the nonmodified tigerinin-1R. Among the analogs, [I10W]tigerinin-1R showed the highest potency with a threshold concentration of 0.01 nM for stimulation of insulin release from the BRIN-BD11 cells. When the [S4R]tigerinin-1R (75 nmol/kg body weight) was treated in combination with glucose to HFD-fed mice, during 60 min of post-administration, it increased the plasma insulin level and reduced blood glucose concentration.

### 2.3. Magainin-AM1 and AM2

Magainin-AM1 and AM2 are both 23-mer peptides first isolated from the skin secretion of the Volcano clawed frog, *Xenopus amieti* [26,39]. These peptides possess weak anti-microbial, anti-inflammatory, low hemolytic activity and hypoglycemic activity. The mechanism for the insulinotropic action involved cell membrane depolarization and an increase in intracellular calcium concentration, but the K_ATP_ channel-dependency is yet not proven. For the magainin-AM2, insulin secretion was also confirmed in BRIN-BD11 beta cells and isolated mice pancreatic islets. In addition to the direct stimulation of insulin release, both magainins could also induce GLP-1 secretion at above 300 nM in GLUTag cells, with no obvious deleterious effects on the cells up to 3 μM [26]. The twice-daily treatment for 15 days of magainin-AM2 (at a dose of 75 nmol/kg) in HFD-fed mice produced a significant improvement in glucose tolerance, insulin sensitivity, and improved beta cell functions [40].

### 2.4. Hymenochirin 1B

Hymenochirin-1B (Hym-1B) was first isolated from the skin secretions of the *Hymennochirus boettgeri* also known as the Congo dwarf clawed frog [28,41]. Similar to the esculentin peptides, the Hym-1B displayed moderate anti-microbial activity and relatively weak hemolytic activity against human erythrocytes (LC_50_: 210 µM) [42]. Regarding the insulinotropic activity of Hym-1B, when tested on BRIN-BD 11 cells, the threshold concentration was 1 nM and the maximum non-toxic stimulatory concentration was 1 μM (1000-fold higher than the threshold) [28]. Like other toxin-derived anti-diabetic peptides, increasing the cationicity of hymenochirin-1B by substitutions of several amino acids (Pro5, Glu6, and Asp9) with Lys showed an enhanced insulinotropic activity. However, most of the tested analogs also showed similar or increased toxicity levels, except for [P5K]Hym-1B and [D9k]Hym-1B. Specifically, the [P5K]Hym-1B showed the highest potency with the lowest cytotoxicity. The threshold concentration was 0.03 nM and the maximum non-toxic stimulatory concentration was 3 μM. Mechanistic studies for the [P5K]Hym-1B revealed interesting results. It appeared that the [P5K]Hym-1B could stimulate insulin release from the pancreatic beta cells by the K_ATP_ channel-independent pathway (with downstream signaling via the PKA pathway). Interestingly, similar to the magainin-AM2 and tigerinin-1R, the [P5K]Hym-1B could also release GLP-1 from the GLUTag cells; suggesting that the stimulation of GLP-1 release from the L cells may also partly explain its insulinotropic activity. Both the [P5K]Hym-1B and [D9k]Hym-1B were available to lower blood glucose and increase the plasma insulin level shortly after i.p. administration in HFD-fed mice. In a related study, a long-term (28 days) treatment with [P5K]Hym-1B of HFD-fed mice (a twice daily administration of 75 nmol/kg by i.p. injection) was carried out and the results showed a significant reduction of blood glucose level with a higher plasma insulin level, improved glucose tolerance, and insulin sensitivity [42].

### 2.5. Alyteserin-2a

Alyteserin-2a is a cationic and amphipathic peptide first isolated from the skin secretions of the midwife toad Alytes obstetricans [29]. The alyteserin-2a possesses anti-microbial and anti-diabetic activity with low hemolytic activity (LC_50_: 135 uM). It could stimulate insulin release from the BRIN-BD11 cells at concentrations above 30 nM. The mechanism for the insulinotropic activity involved membrane depolarization and increased intracellular Ca^2+^ concentration. A group of amino acid-modified analogs have been synthesized and characterized. Opeolu et al. reported that increasing the cationicity of the peptide by 1 or 2 amino acid substitutions at the position of Gly11, Ser14, Asn15 to Lys improved the potency of the insulinotropic action 300 to 1000-fold [43]. However, increasing the hydrophobicity by amino acid substitution at Thr8, Ala9, and Ala10 to Trp decreased the potency.

### 2.6. Brevinin-2-Related Peptide (B2RP)

B2RP, a 21-mer cationic and α-helical peptide, was first isolated from the skin secretions of mink frog *Lithobates septentrionalis* [30]. The term of the B2RP came from the similarity in its amino acid sequence to the peptides belonging to the brevinin-2 family. The B2RP has been identified for its antimicrobial activity along with its insulinotropic activity. The B2RP can induce significant insulin release from the BRIN-BD11 cells at above 1 μM without causing harm to the integrity of the plasma membrane [44]. The B2RP also showed low hemolytic activity (LC_50_: 70 μM) [30]. According to Abdel-Wahab et al. [44], with amino acid substitution of Asp4 or Leu18 to Lys in the peptide structure of the B2RP (increase of cationicity), the potency of B2RP for insulin release was significantly improved (significant insulin release at above 0.3 μM). However, interestingly, substitution of both the Asp4 and Leu18 to Lys residues did not improve the insulinotropic potency but only resulted in higher toxicity (>1 μM). On other hand, increasing the amphipathicity by substituting Lys16 to hydrophobic Ala showed a reduced potency. The administration of [D4K]B2RP to HFD-fed mice at 100 nmol/kg by i.p. injection elicited a significant increase in the plasma insulin level and significantly improved glucose tolerance [44].

### 2.7. Peptide GlycinE-Leucine-Amide (PGLa)-AM1

PGLa-AM1, a cationic 22-mer peptide with an α-helical structure, was first isolated from the skin secretions of frog Xenopus amieti (Pipidae) [45]. It possesses antimicrobial properties with insulinotropic activity. The PGLa-AM1 induced insulin release from BRIN-BD11 cells at above 100 nM [46] and low hemolytic activity (LC_50_: >1000 μM) [31]. In comparison, more cationic analogs of the PGLa-AM1 (substitution of Ala14 or Ala20 to Lys) showed significantly higher potency in stimulating insulin release from the BRIN-BD11 cells (minimum concentrations for significant stimulation of the insulin release were 10 pM and 30 pM, respectively [46]). Both the [A14K] and [A20K] analogs did not affect the integrity of the plasma membrane up to 3 μM. These two analogs also showed great potency for insulin secretion in isolated mouse pancreatic islets. The mechanism for the insulinotropic activity is yet not clarified. The PGLa-AM1 and the two analogs did not show direct effects on the K_ATP_ channels, but elicited a significant membrane depolarization in BRIN-BD11 cells, accompanied with a significant increase in the intracellular Ca^2+^ concentration [46]. These results suggested a possible involvement of K_ATP_ channel-independent pathway. Furthermore, the [A14K] analog induced a significant increase in the cAMP concentration in BRIN-BD11 cells that indicated possible involvement of even other pathways. When evaluated in vivo, both the [A14K] and [A20K] analogs (at 75 nmol/kg by i.p. injection) produced increased plasma insulin levels and improved glucose tolerance.

## 3. Drug Delivery Challenges for Toxin-Derived Anti-Diabetic Peptides

From the drug delivery point of view, the toxin-derived anti-diabetic peptides commonly encounter critical challenges during their journey to the action sites in the body [47,48]. Broadly, this journey could be considered in two steps: (1) traveling from the administration site to the action tissue (e.g., pancreas) through the blood circulation and (2) entering the designated tissue and moving to (or into) the cells of interest. Regarding the first step of the journey, the peptides used to encounter various enzymes and suffer from proteolytic degradation. Furthermore, because of their small size, these peptides used to become rapidly eliminated through the kidney. Collectively, only a limited number of those peptides would successfully reach the target site [49]. On the other hand, during blood circulation, the peptides could be recognized by the immune system and provoke immunogenicity or cause toxicity in normal cells such as the erythrocytes or the endothelia [50,51]. These adverse effects may limit the dose and frequency of their administration. Presumably, because of these limitations, in many reports, the therapeutic effects of those anti-diabetic peptides could be assessed with their analogs possessing improved plasma stability [52].

Once the peptides could successfully reach their targeted tissues, there yet remain challenges to efficiently bind/internalize the cells of interest. Different from exendin-4 [53], the cellular receptors for most of the reported anti-diabetic peptides derived from the skin secretions of amphibians have not been identified yet. A number of structure–activity analysis studies have raised a different possibility that the insulinotropic action may be initiated by direct internalization of the peptides to the beta cells, which reminds them of the cell-penetrating peptides [38,43,54]. Supporting this hypothesis, in related studies, increasing the cationicity or hydrophobicity of the peptides by appropriate amino acid substitution could have improved the potency, stimulating insulin release-possibly by promoting their membrane binding and internalization [55,56]. Nevertheless, to highly augment the potency of these peptides, it would be necessary to first clarify the underlying mechanism for the insulinotropic action of the peptides and further the investigation of their cell-signaling pathways.

## 4. Pharmaceutical Strategies to Improve the Plasma Half-Lives of Anti-Diabetic Peptides

To overcome the obstacles for the toxin-derived anti-diabetic peptides, various approaches have been adopted. To date, in the literature, a widely attempted strategy to improve stability against proteolytic degradation has been the amino acid substitution [57,58]. Specifically, replacing the L form of amino acid residues to the unnatural D form was proven highly effective in vitro [59]. However, this approach may not be sufficient to elicit the full efficacy of the peptides in the animal models. This is because they would still be eliminated rapidly from the animal body, because of their small size. It was found that efficacy and toxicity issues could also be somewhat addressed by the proper amino acid substitution (e.g., increasing the cationicity or hydrophobicity) [28,34,38]. Nevertheless, unless the plasma circulation time of the peptides is dramatically increased, the anti-diabetic peptides could only elicit very limited therapeutic effects in the body and, even if it is available, multiple treatments would be necessary that could lead to reduced compliance. In this chapter, we would like to introduce the two major strategies that have been successfully applied to increase the plasma half-lives of the exendin-4. The first strategy is the physical shielding of the peptides by coupling them with a large molecule such as polymers, and the other is exploiting the FcRn-mediated recycling by linking the peptides with serum albumins through covalent or noncovalent binding with serum albumins or Fc regions of antibodies. The type of modification of the peptides and their plasma half-lives are summarized in Table 2.

### 4.1. Physical Shielding

**Figure 1:**
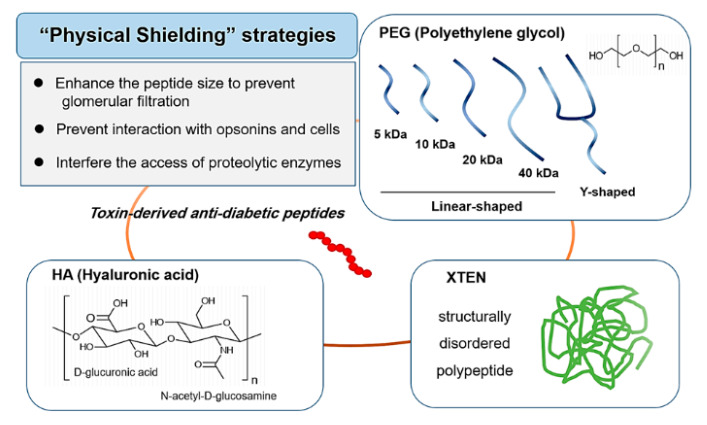
Strategies for physical shielding of anti-diabetic peptides.

Structural modification of small peptides or proteins with attached high molecular weight moieties have proven to be a successful strategy to obtain desirable properties, such as prolonged plasma half-life, enhanced enzymatic stability, and reduced immunogenicity. The underlying mechanism could be explained by two means. One is by enlarging the peptide sizes to avoid their rapid renal clearance, and the other is by providing the peptides with physical shielding that prevents their interaction with opsonins and cells. For this purpose, a wide variety of materials have been adopted that include natural or synthetic polymers, carbohydrates, lipids, and even peptides/proteins. The coupling of these molecules to the peptides was usually achieved by chemical conjugation, but in some cases, genetic engineering was also available (e.g., XTENylation). Regarding the chemical conjugation, the major industrial concerns for production are high heterogeneity in the products, difficulty in maintaining good reproductivity, and relatively high cost. Nevertheless, represented by the success of PEGylation, this strategy has become more popular in both the academic and the industrial field. The scheme of the physical shielding strategies is shown in Figure 1.

#### 4.1.1. PEGylation

“PEGylation” is the process of conjugating polyethylene glycol (PEG) polymer chains to a molecule of interest [73,74]. Specifically, the PEGylation of small biotherapeutics dramatically increases their hydrodynamic radius and allows them to avoid rapid renal clearance [75,76]. The PEG chains also provide a highly hydrophilic hydration shield for the attached molecules that could protect them from opsonization as well as proteolytic degradation. Due to its great effectiveness, to date, the PEGylation technology has been one of the most widely adopted strategies to increase the plasma half-life of macromolecules and indeed a large number of PEGylated drugs have been, to date, clinically available [77]. For the optimal PEGylation of macromolecules, some major considerations will be (1) the size and (2) shape of the PEG chains and (3) the position for PEGylation on the molecules. These factors could not only affect the level of plasma half-life extension but also the intrinsic activity of the attached molecules [78]. Gong et al. prepared an analog of exendin-4, PB-105, which had an amino acid substitution of a serine residue to a cysteine residue at the 39 position [60]. This amino acid substitution little affected the insulinotropic activity of the exendin-4 and moreover allowed site-specific conjugation of a PEG chain via conjugation to the thiol group of the cysteine residue. Exendin-4 conjugates with different sizes (5, 10, 20 and 40 kDa) of linear-shaped PEGs were prepared and, when administered in Sprague-Dawley rats, all the PEGylated exendin-4 showed extended plasma half-lives. Interestingly, conjugation of PEGs with larger molecular weights (from 5, 10, 20 to 40 kDa) showed more extended plasma half-lives. The plasma half-lives of the PEGylated analogs were 6.1, 49.4, 70.5, and 76.4 h for 5 kDa, 10 kDa, 20 kDa, and 40 kDa PEG-conjugated exendin-4 samples, respectively. The results exhibited a linear correlation of the PEG size and the plasma half-life and the AUC (Area under the concentration-time curve). When the 20 kDa PEG-conjugated exendin-4 was administered to STZ (Streptozotocin)-diabetic mice, more extended glucoregulatory activity was observed (t_1/2_: 23 h compared with 5.6 h for the PB-105). Tang et al. also developed a PEGylated exendin-4 by the conjugation of a 20 kDa PEG to the C-terminal of an exendin-4 analog that contained an attached cysteine residue on the 40 position of the peptide [79]. The hypoglycemic effect of the PEG-exendin-4 after single administration on db/db mice was prolonged from 8.4 to 54.9 h. Yun et al. developed a different version of PEGylated exendin-4, named NLY01. The NLY01 was made up of an exendin-4 analog (exendin-4 with C-terminal extension of a cysteine residue at 40 position) conjugated with a Y-shaped 40 kDa-sized PEG [61]. The NLY01 showed a prolonged plasma half-life (38 h in mice, and 88 h in non-human primates) and produced extended pharmacodynamic effects in the tested animal models.

#### 4.1.2. XTEN

XTEN is a structurally disordered anionic polypeptide composed of selected random sequences exclusively made up of amino acids of A, E, G, P, S, and T [80]. The size of the XTEN has been mainly 864 amino acid lengths reported in the literature, but it could also be modified to varying lengths and, interestingly, the length of the XTEN appeared to significantly affect the plasma half-life of the coupled peptides. Like the PEG, the mechanism for the XTEN to improve the blood circulation time for the fused drugs appears to be related to a shielding mechanism. By enlarging the size of the peptides, it prevents them to go through the rapid glomerular filtration and furthermore, physically interferes with the cellular interaction with the endothelial cells and prevents the degradation inside these cells. The reported plasma half-lives of the recombinant fusion protein of exendin-4 and XTEN (E-XTEN) was 12, 32, and 60 h in mice, rats, and monkeys, respectively, and 128 h in humans [62]. Apart from prolonging the plasma half-life, importantly, the XTEN was found to elicit only weak immunogenicity in animals with the presence of adjuvants.

#### 4.1.3. HAylation

Hyaluronic acid (HA) is a naturally existing hydrophilic polymer present in the human body (specifically in the skin) composed of alternating d-glucuronic acid (GlcA) and *N*-acetyl-glucosamine (GlcNAc) units [81]. Possessing great biocompatibility, biodegradability, and viscoelastic properties, HA has long been considered a favorable biomaterial in the fields of medicine and cosmetics. In terms of extending the plasma half-lives of the anti-diabetic peptides, similar to the main idea of PEGylation, the HA-modification could also markedly increase the hydrodynamic size of the peptides [82]. This is because the HA itself has a relatively large size (from 20 kDa to 4000 kDa), and, by absorbing water, could even more highly expand its volume. Kong et al. reported the successful preparation of a long-acting multivalent HA–exendin-4 conjugate using 100 kDa size HA [63]. With the help of the HA shielding, the HA-exendin-4 was proven 20-fold more stable in the serum in vitro, and, in the type 2 diabetic db/db mice model, could elicit hypoglycemic effects for three days by a single subcutaneous injection. Consistently, the histological analysis of the pancreas sections of the tested mice proved greater insulinotropic activity of the HA-exendin-4.

### 4.2. Exploitation of FcRn-Mediated Recycling

**Figure 2:**
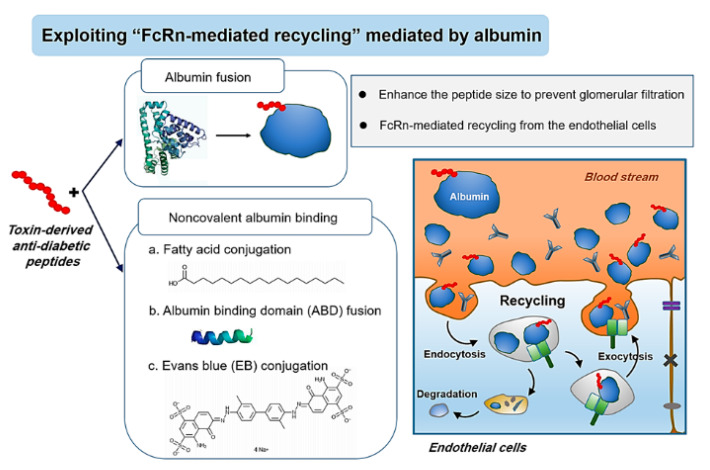
FcRn-mediated recycling of anti-diabetic peptides.

The neonatal Fc receptor (FcRn) is a transmembrane heterodimeric protein possessing a structural similarity to the MHC class I molecules [83]. The history of the FcRn discovery goes back to 1966 when Brambell first proposed the presence of a receptor responsible for the prenatal transport of IgG from mother to fetus across the yolk sac [84]. Later, he also postulated that the same receptor may also play a key role in regulating the serum half-life of IgGs. In 1985, the FcRn was first isolated from the small intestine of a neonatal rat and the corresponding genes were further cloned [85]. Thereafter, the interaction of the FcRn with the Fc region of the IgGs and also with serum albumins was identified [84]. Further studies revealed that FcRn mainly resides in the endosomal compartment of vascular endothelial cells (but is not limited to only these cells) and could bind IgGs and serum albumins in a pH-dependent manner [86]. When circulating IgGs and albumins become endocytosed into endothelial cells, at acidic pH (5–6.5), both could strongly bind to the FcRn. This high-affinity FcRn binding could provide the molecules with a great means of protection from lysosomal degradation. Once rescued, these molecules reach back to the cell membranes and, at a neutral pH, the binding affinities between the FcRn and IgGs and albumins becomes significantly reduced, allowing them to be either recycled back to the blood circulation or transcytosed across the endothelia to the tissues [86]. Thanks to this FcRn-mediated recycling process, the IgGs and serum albumins, which are the most abundant serum proteins, possess markedly longer (several weeks) plasma half-lives than other endogenous proteins. Later, it was also found that coupling peptide or protein drugs to the Fc region of IgGs or albumins could significantly prolong their blood circulation times. Here, we introduced the recent studies exploiting the FcRn-mediated recycling to extend the plasma half-lives of toxin-derived anti-diabetic peptides focusing on albumin-based approaches [87]. The scheme of the FcRn-mediated recycling via albumin binding is shown in Figure 2.

#### 4.2.1. Albumin Fusion

Albumin, produced by hepatocytes, comprises the majority (55%) of the total plasma proteins. Despite the wide variability of the sequence among species, the albumins appear to have similarities in the 3-D structures [88]. In the case of humans, the HSA is a 66.5 kDa sized non-glycosylated protein composed of a single polypeptide chain of 585 amino acids possessing 17 pairs of intramolecular disulfide bonds with one extra free cysteine residue [89]. The HSA consists of thre homologous domains (named DI, DII, and DIII) which are connected with long flexible loops [90]. The HSA binds FcRn at a 1:1 stoichiometry through its domain DIII to the heavy chain region of the FcRn [91]. This binding site of albumin on the FcRn does not overlap with the IgG binding sites, so it does not compete for the FcRn binding with the IgGs. However, the binding affinity between the FcRn and albumin is weaker than the FcRn-IgG [92]. Importantly, the HSA possesses a long plasma half-life of 19 days, which heavily relies on the FcRn-mediated recycling [91]. Like the interactions between the IgG and FcRn, albumin’s FcRn binding is strictly dependent on the pH. Binding occurs at a low pH, but not at neutral pH. As it shares the same FcRn-mediated recycling mechanism, the albumin fusion has also been considered an attractive strategy for improving the pharmacokinetic profiles of small macromolecule drugs.

Albumin fusion could be achieved either by chemical conjugation or genetic recombination. Both methods have their advantages. While the chemical conjugation is relatively convenient and favorable for preparing multivalent conjugates, the genetic recombination approach may be more reproductive and economic for industrial production. Adopting the chemical conjugation method, Kim et al. reported the synthesis of an exendin-4-HSA conjugate coupled via a 5 kDa size PEG chain as the crosslinker [64]. Interestingly, this conjugate (HSA-PEG-Ex4) had a significantly longer plasma half-life (24.2 h) than not only the unmodified exendin-4 (2.1 h) but also the HSA-Ex4 (conjugate without PEG linker; 11.4 h) in ICR mice after i.p. injection. Accordingly, in the db/db diabetic mice model, the hypoglycemic duration of HSA-PEG-Ex4 was greater (31 h) than that of the exendin-4 (7.0 h).

To produce recombinant exendin-4-albumin fusion proteins, eukaryotic hosts such as yeast may be more favorable over the prokaryotic expression systems (e.g., E. *coli*). This is because of the complications of the albumin structure that possesses 17 disulfide bridges. Huang et al. produced a recombinant exendin-4 and HSA fusion protein (named “rEx-4/HSA”) coupled with a GGGGS linker from yeast Pichia pastoris [65]. The rEx 4/HSA showed a long plasma half-life (77.4 h) in cynomolgus monkeys. Similarly, Li et al. also reported the development of an exendin-4/HSA fusion protein coupled by the GGGGS peptide linker [66]. The difference was that they developed a tandem dimeric exendin-4 and albumin fusion protein (named “E2HSA”). The E2HSA showed a plasma half-life of 54 h in healthy rhesus monkeys and elicited a significant reduction in the blood glucose level in the monkeys for a prolonged duration (at least for 42 h).

#### 4.2.2. Non-Covalent Albumin Binding

In the blood, albumins serve as carrier proteins for various substances, such as hormones, steroids, and fatty acids [93]. Specifically, albumin possesses a total of nine binding sites for fatty acids distributed throughout the albumin molecule. Fatty acid binding spontaneously occurs in the plasma and, while circulating in the bloodstream, slowly dissociates [94,95]. Exploiting this reversible but stable fatty acid-mediated albumin binding, many research attempts have been pursued to extend the plasma half-lives of macromolecules by conjugation with fatty acids. To date, the best example that accomplished this goal was the liraglutide (Victoza^®^ by Novo Nordisk) which is a clinically approved long-acting analog of glucagon-like peptide-1 agonist (GLP-1) conjugated with a myristic acid (at the N-terminal lysine position). The plasma half-life of liraglutide in humans is about 11–15 h, which allows once-daily injection for sufficient glucoregulation.

Fatty acid conjugation was also tried for exendin-4. On the exendin-4 peptide, there are a total of three free amine groups available for chemical conjugation (at the positions of N-terminal, Lys12, Lys 27). Chae et al. synthesized lithocholic acid-exendin-4 conjugates with site-specific conjugation of lithocholic acid at different lysine residues: Lys12 (LCA-M2), Lys 27 (LCA-M1), or both Lys12 and Lys 27 (LCA-Di) [67]. Through in vitro binding and insulinotropic activity assays, out of the three derivatives, the LCA-M1 was found to possess the highest GLP-1R binding affinity and insulinotropic activity. The K_d_ values of the LCA-M1 was 0.47 nM (exendin-4, LCA-M2, and LCA-Di: 0.14, 0.55, and 71.7 nM, respectively) and the EC_50_ for insulinotropic activity in pancreatic islets was 8.1 nM (exendin-4, LCA-M2, and LCA-Di: 4.1, 13.9, and 27.4 nM, respectively). The LCA-M1 showed a plasma half-life of 9.7 h in Sprague-Dawley rats (exendin-4: 1.6 h) and the hypoglycemic effects in C57BL/6 db/db mice prolonged for more than 24 h with as low as 15 nmol/kg dose. Lee et al. reported that starting from the N-terminal, amino acids 1–33 are the minimum motif required for anti-diabetic activity and stability [68]. With the exendin-4 (1–33), they substituted Ser33 with Lys for acylation and conjugated with different lengths of fatty acids. Data revealed that with the increasing size of the fatty acid, albumin binding tends to increase, but could give more interference to the GLP-1R binding of the exendin-4. The in vitro plasma stability test results showed that compared with the unmodified exendin-4 (7.6 h), the degradation half-lives of valeric(C5:0)-, capric(C10:0)-, and palmitic(C16:0)-acylated exendin-4(1–33) were extended to 12.4, 24.2, and 28.4 h, respectively. The plasma half-life of capric acid-conjugated exendin-4 analog (Ex-4(1–32)K-Cap) was 2.25 h and, when administered subcutaneously, the blood glucose-lowering effects maintained for over 40 h at 5 nmol/kg dose in C57/BL6 db/db mice. Vasu et al. reported octanoic acid-modified esculentin-2CHa analogs ([Lys^15^-octanoate]-esculentin-2CHa(1–30) and [Lys^23^-octanoate]-esculentin-2CHa(1–30)) [23]. Both the lipid-conjugated esculentin-2CHa analogs showed slightly improved in vitro plasma stability (62% and 79% degradation compared with 93% by esculentin-2CHa(1–30) after 8 h in mice plasma). In further studies, specific pharmacokinetic data are not presented, but chronic treatment (twice daily administration for four weeks of the [Lys^15^-octanoate]-esculentin-2CHa(1–30) at 75 nmol/kg) to normal NIH swiss mice provided significant lowering of blood glucose and cholesterol levels [34]. Advancing the albumi-binding strategy, Lee et al. combined the nanotechnology to further extend the plasma half-lives of exendin-4. In their study, decanoic acid-modified glycol chitosan (DA-GC) hydrogels containing palmitic acid-modified exendin-4(Ex4-C16) were prepared [69]. The Ex4-C16 containing DA-GC hydrogels (Ex4-C16 DA-GC) could slowly release the EX4-C16 for 21 days in vitro and, with s.c. administration in db/db diabetic mice, hypoglycemic effects were achieved for eight days.

Another way to achieve the noncovalent albumin binding is to utilize albumin-binding proteins/peptides such as albumin-binding domains (ABDs) and albumin-binding peptides [96]. The ABDs, derived from natural sources like streptococcal protein G, have relatively small sizes (about 40–60 amino acids length) and are capable of binding albumins with good specificity and affinity [97]. Therefore, by fusing the ABDs to therapeutic peptides, it is available to enlarge the size of their peptides sufficient to escape the glomerular filtration and further exploit the FcRn-mediated recycling process. Zhong et al. prepared an exendin-4 derivative (named LEx4) composed of an albumin-binding domain (ABD) fused to a native exendin-4 [70]. Lex4 showed high-affinity binding with rat and monkey serum albumins (K_a_: 1.26 × 10^6^ M^−1^, and 1.52 × 10^6^ M^−1^, respectively), and the albumin binding provided improved stability against proteolytic degradation in plasma (t_1/2_: 4.81 h vs. 7.31 h in the presence of rat serum albumin). In Sprague-Dawley (SD) rats, LEx4 showed extended plasma half-life (3.3-fold; plasma half-life: 2.4 h vs. 8.1 h after s.c. injection) than exendin-4, and, accordingly, prolonged antidiabetic effects in type 2 diabetic mice. The duration of the antidiabetic effects for the LEx4 group was 1.9-fold—longer than that of the exendin-4 group (8.7 h vs. 4.7 h). Levy et al. also reported the synthesis of an exendin-4 and ABD fusion protein [71]. The exendin-4-ABD (named “ABD035”) showed a picomolar affinity toward the human serum albumin (K_D_: 4.9 pM) and a circulating plasma half-life of 16 h in Sprague-Dawley (SD) rats after intravenous injection. Moreover, in cynomolgus monkeys, its plasma half-life was estimated to be 11–13 days (by i.v. injection). In the same literature, they also reported the activity of the hybrid peptide composed of exendin-4 and an albumin-binding peptide (ABP). The ABP adopted in the study was discovered by Dennis et al. and named SA21 [98]. The SA21 was an 18-mer peptide and possessed a good binding affinity (K_D_: 467 nM) to human albumin (also to rat and rabbit albumins). The exendin-4-ABP showed a plasma half-life of 11 h in the rats (by i.v. injection). When comparing the efficacy with the exendin-4-ABD, the exendin-4-ABP possessed a 100-fold higher potency than the exendin-4-ABD in the GLP1R functional assay, but the binding affinity to the albumin was 312,000-fold lower. Presumably, due to this difference in the albumin-binding affinity, the exendin-4-ABP showed a shorter blood circulation time than the exendin-4-ABD. The difference of the plasma behavior was more obvious in the monkey test, where the exendin-4-ABP exhibited a drastic reduction of the plasma concentration level within one day.

Apart from the lipid-binding sites, the serum albumins also possess two specific binding sites for varied small molecules, named Sudlow sites I (warfarin–azapropazone binding site) and II (benzodiazepine binding site). Specifically, the site I is known to interact with aromatic carboxylic acids that have a peripheric negative charge. A representative molecule that binds with good affinity to this site 1 is the Evans blue (EB) which is a synthetic bis-azo dye. The capacity of EB binding has been estimated to be 8–14 moles per albumin molecule [99]. Because of its avid albumin-binding ability, the EB has been of interest as an albumin-binding ligand. Recently, Chen research group reported an exendin-4 and a truncated Evans Blue conjugate (named “Abextide”), prepared by chemical conjugation via maleimide-tEB and exendin-4 with a Cys40 [72]. The Abextide showed equivalent GLP1R binding ability to exendin-4, but an extended biological half-life (35 h; 7-fold) in Balb/c mice, and prolonged hypoglycemic effects (36 h; 3.6-fold) in db/db diabetic mice. Furthering the study of Abextide, Niu et al. carried out a pharmacodynamics study in diabetic Cynomolgus monkeys [100]. The results showed that the Abextide could elicit similar levels of hypoglycemic activity with albiglutide, a clinically approved long-lasting GLP-1 agonist, and exerted hypolipidemic and anorexic activities.

## 5. Conclusions

Many toxin-derived peptides found from skin secretions of amphibians have shown great potential to be developed as anti-diabetic drugs. These peptides could stimulate insulin release from the beta cells and lower blood glucose levels. Despite their potent hypoglycemic activity, the mechanism for the insulinotropic action has not been elucidated yet and, more importantly, most of these peptides could produce only low in vivo activity because of their short blood circulation time. This was also the case for exendin-4 which is now clinically available with longer acting derivatives. This has been realized by the development of various strategies that could effectively increase its plasma stability and blood circulation time. Among various strategies explored to date, two major strategies have gained the most success. They are (1) physical shielding by coupling with high molecular weight moieties, and (2) exploiting the FcRn-mediated recycling by binding serum albumin or the Fc region of antibodies. These approaches have especially gained great success in extending the plasma half-life of exendin-4 (from several hours to several days). However, there are very limited reports of studies applying any of these strategies to the anti-diabetic peptides derived from the skin secretions of amphibians. The explored strategies might not fit universally to all the peptides as it did to the exendin-4. However, as they share many structural similarities, it is not too wild to predict that they would not be helpful. Indeed, as shown by the successful application of the fatty acid-conjugation of esculentin-2CHa(1–30), there might be strategies that fit well with those anti-diabetic peptides. Of course, a great effort would be required to search for the optimal strategy of the interested peptides.

## Figures and Tables

**Table 1:** Toxin-Derived Anti-diabetic Peptides.

Peptide	Sequence	Threshold Concentration for Insulin Release from Beta Cells (nM)	Hemolytic Activity (LC_50_; μM)	Reference
Esculentin-2CHa	GFSSIFRGVAKFASKGLGKDLAKLGVDLVACKISKQC	100	150	[23,24]
Tigerinin-1R	RVCSAIPLPICH	0.1	500	[25]
Magainin–AM1	GIKEFAHSLGKFG KAFVGGILNQ	-^1^	-	[26]
Magainin–AM2	GVSKILHSAGKFGKAFLGEIMKS	-	>100	[27]
Hymenochirin-1b	IKLSPETKDNLKKVLKGAIKGAIAVAKMV	1	210	[28]
Alyteserin-2a	ILGKLLSTAAGLLSNL	30	135	[29]
Brevinin-2-related peptide (B2RP)	GIWDTIKSMGKVFAGKILQNL	1000	-	[30]
Brevinin-2-related peptide (B2RP)	GMASKAGSVLGKVAKVALKAAL	100	-	[31]

^1^ The symbol indicates that data are not available.

**Table 2:** Type of Modification Applied for Extension of the Plasma Half-Life of Exendin-4.

Type of Modification	Plasma Half-Life (Tested Animal Species, Administration Route)	Duration of Hypoglycemic Action (Tested Animal Species)	Reference
PEGylation with different sizes (5, 10, 20, and 40 kDa) of linear PEGs	6.1 to 76.4 h (rat, *i.v.* (intravenous))	t_1/2_: 23 h (for 20 kDa PEG conjugated exendin-4) (mouse)	[60]
PEGylation with Y-shaped 40 kDa PEG	38 h (mouse, *s.c.* (subcutaneous)) 88 h (monkey, *s.c.*)	-^1^	[61]
XTENylation	12 h (mouse) 32 h (rat) 60 h (monkey, *s.c.* and *i.v*.) 128 h (human)	-	[62]
Hyaluronic acid (HA) conjugation	-	3 days (mouse)	[63]
Human serum albumin (HSA) chemical conjugate via PEG linker	24.2 h (mouse, *i.p.* (intraperitoneal))	31 h (mouse)	[64]
HSA fusion	56.7 h (monkey, *i.v.*) 77.4 h (monkey, *s.c.*)	-	[65]
HSA fusion to tandem dimeric exendin-4	54 h (monkey, *s.c*.)	42 h (monkey)	[66]
Lithocholic acid conjugate	9.7 h (rat, *s.c*.)	≥24 h (mouse)	[67]
Capric acid conjugate of exendin-4 analog (Ex-4(1-32) K-Cap)	2.25 h (mouse, *s.c*.)	≥40 h (mouse)	[68]
Decanoic acid-modified glycol chitosan hydrogel encapsulation (DAGC-Exendin-4-C16)	-	8 days (mouse)	[69]
Albumin-binding domain (ABD) fusion	8.1 h (rat)	8.7 h (mouse)	[70]
ABD035 fusion	16 h (rat, *i.v*.)	-	[71]
Albumin-binding peptide (ABP; SA21) fusion	11 h (rat, *i.v*.)	-	[71]
Truncated Evans blue (tBE) conjugation	35 h (mouse, *s.c*.)	36 h (mouse)	[72]

^1^ The symbol indicates that data are not available.
